# Therapeutic potential of plant iridoids in depression: a review

**DOI:** 10.1080/13880209.2022.2136206

**Published:** 2022-10-27

**Authors:** Yaoyao Kou, Zhihao Li, Tong Yang, Xue Shen, Xin Wang, Haopeng Li, Kun Zhou, Luyao Li, Zhaodi Xia, Xiaohui Zheng, Ye Zhao

**Affiliations:** a Three level Scientific Research Laboratory of National Administration of Traditional Chinese Medicine, Northwest University, Xi’an, PR China; b Key Laboratory of Resource Biology and Biotechnology in Western China, Ministry of Education, Northwest University, Xi’an, PR China

**Keywords:** Secoiridoids, mental disorders, molecular mechanisms

## Abstract

**Context:**

Depression is a mental disorder characterized by low mood, reduced interest, impaired cognitive function, and vegetative symptoms such as sleep disturbances or poor appetite. Iridoids are the active constituents in several Chinese classical antidepressant formulae such as Yueju Pill, Zhi-Zi-Hou-Po Decoction, Zhi-Zi-Chi Decoction, and Baihe Dihuang Decoction. Parallel to their wide usages, iridoids are considered potential lead compounds for the treatment of neurological diseases.

**Objective:**

The review summarizes the therapeutic potential and molecular mechanisms of iridoids in the prevention or treatment of depression and contributes to identifying research gaps in iridoids as potential antidepressant medication.

**Methods:**

The following key phrases were sought in PubMed, Google Scholar, Web of Science, and China National Knowledge Internet (CNKI) without time limitation to search all relevant articles with *in vivo* or *in vitro* experimental studies as comprehensively as possible: (‘iridoid’ or ‘seciridoid’ or ‘depression’). This review extracted the experimental data on the therapeutic potential and molecular mechanism of plant-derived iridoids for depression.

**Results:**

Plant iridoids (i.e., catalpol, geniposide, loganin), and secoiridoids (i.e., morroniside, gentiopicroside, oleuropein, swertiamarin), all showed significant improvement effects on depression.

**Discussion and conclusions:**

Iridoids exert antidepressant effects by elevating monoamine neurotransmitters, reducing pro-inflammatory factors, inhibiting hypothalamic-pituitary-adrenal (HPA) axis hyperactivity, increasing brain-derived neurotrophic factor (BDNF) and its receptors, and elevating intestinal microbial abundance. Further detailed studies on the pharmacokinetics, bioavailability, and key molecular targets of iridoids are also required in future research, ultimately to provide improvements to current antidepressant medications.

## Introduction

The Diagnostic and Statistical Manual for Mental Disorders defines a mental disorder as a syndrome characterized by clinically significant impairments in an individual’s cognition, emotional regulation, or behaviour, which reflects potential psychological, biological, or developmental dysfunction (Stein et al. 2021). Common mental disorders include depression, anxiety disorder, bipolar disorder, and schizophrenia. A study by Mendenhall et al. (2017) shows that more than 17.5% of Chinese adults suffer from mental disorders, with depression accounting for the majority. Depression is estimated to be the world’s largest burden of disease by 2030 and the largest contributor to non-fatal health loss worldwide (Varghese et al. 2021). The major causes of depression are considered to be genetic susceptibility and environmental influences. Changes in the neuroendocrine system, immune system, and metabolic level caused by stress may be the pathological basis of depression. Under chronic stress, the HPA axis activity is enhanced, resulting in an increase in glucocorticoid (GC) secretion, thereby affecting the release of pro-inflammatory cytokines. Pro-inflammatory cytokines suppress the composition of the blood-brain barrier (BBB) as well as the expression of associated proteins and factors such as occludin, zonula occludens (ZO), tight junction protein 5, and junctional adhesion molecule (JAM), resulting in increased BBB permeability (Meaney 2001; Xu et al. 2013; Xia et al. 2017). The increased permeability of the BBB in turn promotes immune cells and pro-inflammatory cytokines to enter the brain (Zrzavy et al. 2019). Furthermore, high levels of pro-inflammatory cytokines promote the production of neurotoxic products via the kynurenine pathway, ultimately producing neurotoxic effects on specific brain regions (Won and Kim 2016).

Medications are the major treatment for depression. There are five main classes of antidepressants commonly used today: selective serotonin reuptake inhibitors (SSRIs), selective serotonin-norepinephrine reuptake inhibitors (SNRIs), tricyclic antidepressants (TCAs), monoamine oxidase inhibitors (MAOIs), and atypical antidepressants (Skånland and Cieślar-Pobuda 2019). Outpatient-related reports state that only 25% of patients are in remission within 6 months and over 50% of patients are still depressed after 2 years. The chance of relapse is high, with approximately 80% of patients relapsing at least once in their lifetime (Wells et al. 1992; Penninx et al. 2011). In addition, these medications have a variety of negative effects, such as nausea, dizziness, headaches, and gastrointestinal discomfort, which can also lead to weight gain, sexual dysfunction, and sleep disorders (Cassano and Fava 2004; Cipriani et al. 2018). As a result, pharmaceutical practitioners pay attention to the screening and discovery of beneficial ingredients from natural products for the treatment of depression with few side effects.

**Figure 1.**
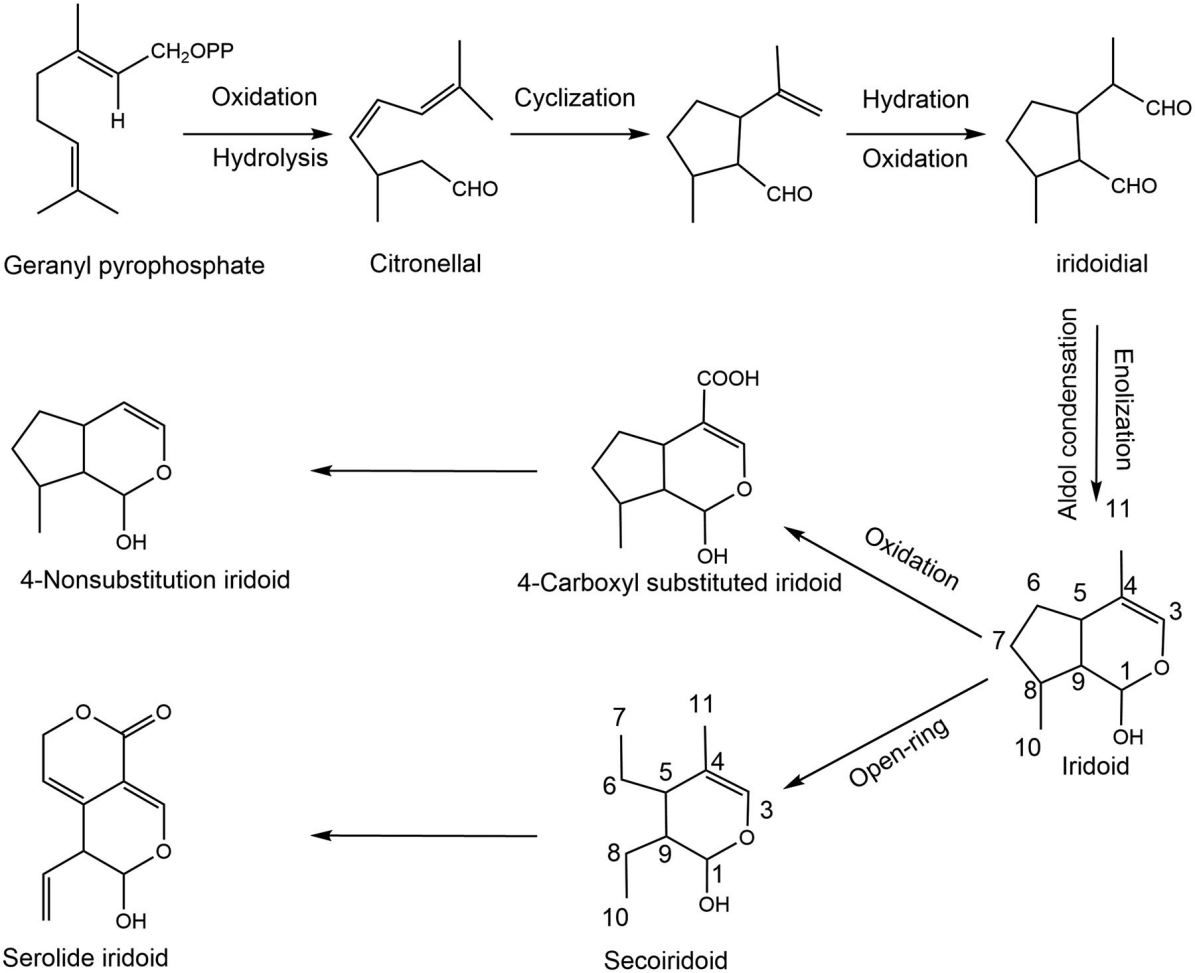
Biosynthesis pathway of iridoids.

In China, classical compound formulae such as Yueju Pill (Zhang H et al. 2015), Zhi-Zi-Hou-Po Decoction (Yao et al. 2013), Zhi-Zi-Chi Decoction (Qu et al. 2014), Baihe Dihuang Decoction (Miao et al. 2019), and other classical prescriptions, have been used to treat depression for thousands of years. Some classical formulae are shown in Table 1. Among them, iridoid compounds with a methylcyclopentane-[C]-pyran skeleton structure are a class of natural lipophilic compounds with endogenous neurotrophic factors, which are considered potential lead compounds for the treatment of neurological diseases and are the active ingredients in the above antidepressant formulae (Habtemariam 2018). Iridoids are a class of naturally occurring monoterpenoids, which are the acetal derivatives of iridodial. Iridoids, isolated from the secretions of the Argentine ant *Iridomyrmex humilis* (Mayr) in 1925, were the first antibiotic found in animals and widely distributed in plant families of Scrophulariaceae, Rubiaceae, Labiatae, Loganaceae, Gentianaceae, and Oleaceae. Based on their structure iridoids are divided into two basic skeletons, iridoids and secoiridoids, which are mainly found in plants as glycosides due to the instability of the C1-OH group of the hemiacetal. The biosynthesis pathway is shown in Figure 1. In recent years, more than 800 types of iridoid compounds have been isolated and identified from plants by liquid chromatography-mass spectrometry (LC-MS) and nuclear magnetic resonance (NMR), most of which are glycosides, and the non-glycoside iridoid compounds are only found in 60 species, and the secoiridoid compounds are found in 30 species (Guo et al. 2011). Modern pharmacology reveals that iridoid compounds have potentiating activities involving anti-inflammation, antioxidation, immunomodulation, neuroprotection, and nerve growth factor (Dinda et al. 2019).

**Table 1.** Some classical formulae associated with depression.

Preparation name	Composition	Iridoid compounds	References
Zhi-Zi-Chi Decoction	*Gardenia jasminoides, fermented soybean*	geniposide	(Qu et al. 2014)
Zhi-Zi-Hou-Po Decoction	*Gardenia jasminoides, Magnolia officinali, Citrus aurantium*	geniposide	(Yao et al. 2013)
Danzhi-Xiaoyao-San	*Bupleurum chinense, Angelica sinensis, Paeonia lactiflora, Poria coco, Atractylodes macrocephala, Gardenia jasminoides, Paeonia suffruticosa*	geniposide	(Wang et al. 2022)
Baihe Dihuang Decoction	*Lilium landfolium, Rehjnannia glutinosa*	catalpol	(Miao et al. 2019)
Yueju Pill	*Cyperus rotundus , Ligusticum chuanxiong, Atractylodes lancea*	geniposide	(Zhang et al. 2015)
Longdan Xiegan Decoction	*Gentiana manshurica, Scutellaria baicalensis, Gardenia jasminoides, Alisma orientate, Akebia quinata, Plantago asiatica, Angelica sinensis, Rehjnannia glutinosa, Bupleurum chinense, Glycyrrhiza uralensis*	gentiopicroside, geniposide	(Wan 2017)
Liuwei Dihuang Pill	*Rehjnannia glutinosa, Cornus officinalis, Paeonia suffruticosa, Dioscorea opposita, Poria coco, Alisma orientat*	catalpol, loganin, morroniside	(Liu et al. 2017)
Jingui Shenqi Pill	*Rehjnannia glutinosa, Poria coco, Dioscorea opposita, Cornus officinali, Paeonia suffruticosa, Alisma orientate, Cinnamomum cassia, Achyranthes bidentata, Plantago asiatica, Aconitum carmichaelii*	loganin, morroniside, catalpol	(Li et al. 2019)
Xuefu Zhuyu Decoction	*Prunus persica, Achyranthes bidentata, Angelica sinensis, Rehjnannia glutinosa, Crocus sativus, Ligusticum chuanxiong, Paeonia ladiflora, Platycodon grandiflorum, Bupleurum chinense, Citrus aurantium, Glycyrrhiza uralensis*	catalpol	(Wang et al. 2021)

Several iridoid compounds generated from natural products have been reported to treat depression and other mental diseases (Li, Wang, et al. 2020). For example, catalpol, an iridoid glycoside extracted from the roots of *Rehmannia glutinosa* (Gaertn.) Libosch. ex Fisch. & C.A.Mey. (Scrophulariaceae), improves cognitive impairment and therefore treats depression (Cui et al. 2004). Geniposide, as the largest quantity of iridoid chemicals in *Gardenia jasminoides* Ellis (Rubiaceae), is one of the most effective components in the treatment of depression (Chen G et al. 2021). This paper systematically reviews fundamental researches on the treatment of depression with iridoid compounds, highlighting their neuroprotective potential in the prevention and treatment of depression, and focussing on the pathway targets and mechanisms of action, ultimately presenting the future applications of iridoid compounds as highly effective antidepressant drugs with few side effects.

## Depression

In the 1750s, Samuel Johnson first associated ‘depression’ with low mood (Rousseau 2000). Subsequent studies have defined depression as a chronic mental disorder characterized by low mood, lack of interest, slowed thinking, and a sense of worthlessness and guilt. Depression is often accompanied by loss of appetite, insomnia, poor concentration, weight loss, and increased fatigue (Nemeroff 2007; Krishnan and Nestler 2008). In addition, cognitive functions, including learning, memory, and attention, are disrupted (Ogren et al. 2008). Depression also raises the risk of diseases including diabetes, heart disease, and stroke, adding to the disease load (Whooley and Wong 2013). Depression is classified according to its severity into three types: mild depression, moderate depression, and major depressive disorder (MDD). Over 350 million people worldwide suffer from depression, and the number of individuals suffering from depression rises every year. Women are significantly more likely to suffer from depression than men, with postpartum depression being the most common type (Parker et al. 2014). While hereditary factors account for one-third of the risk of depression, environmental influences are considered to account for the other two-thirds (Geschwind and Flint 2015). The recent discovery of new *N*-methyl-d-aspartate receptor (NMDA-R) antagonists, represented by Spravato (esketamine), became the first antidepressant with a new mechanism to be approved by the FDA. Yet, this drug is highly controversial in terms of addressing long-term effectiveness, risk of side effects, and long-term abuse (Katalinic et al. 2013; Zanos et al. 2018). As a result, new effective antidepressant drugs are required.

### Pathophysiological mechanism and biomarkers of depression

**Figure 2.**
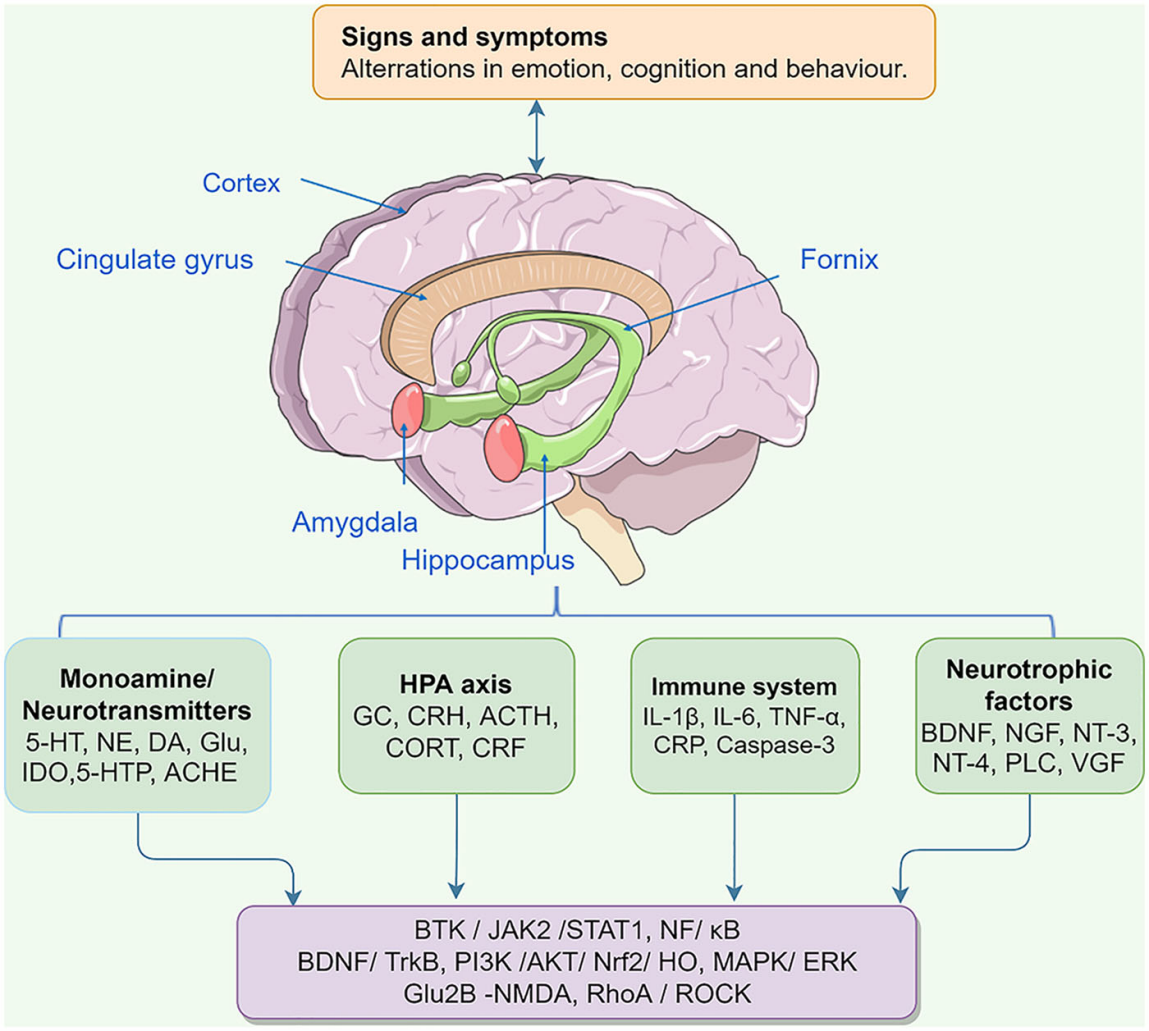
The aetiology and pivotal molecular targets of depression. Glu, Glutamate; ACTH, adrenocorticotropic hormone; CORT, corticosterone; VGF, vascular growth factor.

Depression is a highly heterogeneous mental disorder whose pathogenesis is usually accompanied by reduced levels of monoamine neurotransmitters, elevated inflammatory factors, disturbances in the neuroendocrine system, reduced levels of neurotrophic factors, and reduced abundance of gut microflora. The aetiology and pivotal molecular targets of depression are shown in Figure 2.

#### Reduced monoamine neurotransmitters

The monoamine hypothesis suggests that depression is caused by the decline of monoamine neurotransmitter concentration or function in the synaptic cleft of the central nervous system (CNS) (Castren 2005). As early as the 1970s, lower concentrations of monoamine neurotransmitters such as 5-hydroxytryptamine (5-HT), dopamine (DA), and norepinephrine (NE) were recognized to cause depression (Coppen et al. 1965; Schildkraut et al. 1965; Andersen et al. 1975). Most antidepressants currently used in the clinic, such as TCAs, MAOIs, and SNRI, are targeted to enhance the function of neurotransmitters 5-HT and NE systems by inhibiting the reabsorption or degradation of synaptic monoamine transmitters to increase the concentration of synaptic transmitters to achieve antidepressant effects. The monoamine doctrine, on the other hand, has severe limitations in that targeted monoamines activate the neurotransmitter system within hours of administration, whereas antidepressant effects often take 2–6 weeks to manifest (El Mansari et al. 2010). It is now believed that alterations in brain gene expression that are elicited after chronic treatment might underlie the effects of antidepressants (Wong and Licinio 2001). As a result, the monoamine theory of depression appears to be oversimplified.

#### Disturbances in the neuroendocrine system

The HPA axis is central to an integrated neurobiological model that seeks to explain the long-term consequences of early trauma. A series of animal studies have shown that abnormal adrenocorticotropic hormone (ACTH) activity in patients with depression, which has also been confirmed in a clinical study where patients with depression often have hyperfunction of the HPA axis, causing an abnormal release of anterior pituitary ACTH, GC and corticotrophin-releasing hormone (CRH) (Meaney 2001; Meltzer et al. 2010). A clinical investigation reveals that individuals who have been sexually or physically abused as children have significantly increased HPA axis activity when exposed to standardized psychosocial stressors or following endocrine tests that seek to suppress HPA activity as adults (Stetler and Miller 2011). Furthermore, the HPA axis is one of the most researched biological systems in MDD. Cortisol levels were shown to be increased in MDD patients in a meta-analysis. HPA alterations were linked to impaired cognitive function in these patients, and were more common and pronounced in MDD patients with melancholic and/or psychotic characteristics, as well as in older MDD patients (Hinkelmann et al. 2009; Stetler and Miller 2011). Therefore, deeper clinical and biological phenotyping of depressed patients will lead to the discovery of depression subtypes of patients who are more likely to react to a certain HPA axis treatment.

#### Reduced neurotrophic factors

Neurotrophic factors include brain-derived neurotrophic factor (BDNF), nerve growth factor (NGF), and the neurotrophin-3 (NT-3) and neurotrophin-4/5 (NT-4/5). BDNF is involved in neuroplasticity and repair of stress-related neurological damage, it has been reported to be the most researched neurotrophic factor in the field of depression-related neurobiology (Park and Poo 2013). Clinical studies have shown that low levels of BDNF can be observed in the hippocampus and prefrontal cortex (PFC) of patients with MDD, with atrophy of the hippocampus, neuronal apoptosis, and loss of synapses in the brain (Duman and Monteggia 2006). Analysis protein of BDNF levels in blood of depressed patients also reveals low levels of BDNF in their serum (Jiang et al. 2017). Additionally, plasma levels of BDNF appear to be more significantly reduced in patients with MDD who are intending to commit suicide compared to those with minor depression (Kim et al. 2007). Antidepressant medication or intracranial stimulation of increased BDNF expression may alleviate depressive symptoms in patients (Duclot and Kabbaj 2015). Following that discovery, BDNF was observed to interact with its receptor, tyrosine kinase receptor B (TrkB) to set off a chain of events playing a crucial role in the pathophysiology of depression. TrkB phosphorylation promotes cell survival, growth, and differentiation by regulating the phosphatidylinositol-3 kinase/protein kinase B (PI3K/AKT) pathway, the mitogen-activated protein kinase/extracellular regulated protein kinases (MAPK/ERK) pathway, and the phospholipase γ/inositol triphosphate (PLCγ/IP3) intracellular signalling pathway (Park and Poo 2013).

#### Lower abundance of gut microflora

Gut microbiota has been demonstrated to play a unique role in complicated brain illnesses including depression (Liang et al. 2018). The abundance and diversity of gut microbiota in patients with depression were considerably lower than those in healthy controls, according to a study (Kelly et al. 2016). Faeces from patients with depression were transplanted to sterile mice, and mice developed behaviours related to depression such as mania and anxiety, and physiological characteristics such as tryptophan metabolism changed. Depression can alter the gut microbiota and affect the host’s metabolic phenotype, according to research on chronic variable stress rats using 16SrRNA gene sequencing and metabolomics (Yu et al. 2017). By performing Genome Shotgun Sequencing of faecal samples from patients with depression and healthy people after human clinical practice, Lai et al. (2021) discovered that gut microbiota may be involved in the pathogenesis of depression and that the change in gut microbiota may be a biomarker for distinguishing patients with depression and healthy people. However, not all gut microbiota is detrimental to mood and cognitive processes. For example, probiotics supplementation improves cognitive performance and mood in healthy adults (Tillisch et al. 2013; Marotta et al. 2019). Also, short-chain fatty acids produced from indigestible fibres induce microglia maturation and activation, and BDNF production (Sandberg et al. 2018). A thorough investigation of the brain-gut axis in depression is expected to shed light on the disease’s pathophysiological mechanism.

#### Elevated inflammatory factors

Smith (1991) revealed that cytokines like interleukin-1β (IL-1β) and interleukin-6 (IL-6) might cause hyperactivity of the HPA axis and impaired 5-HT metabolism, leading to depression. Meta-analyses confirmed that serum levels of pro-inflammatory cytokines such as C-reactive protein (CRP), IL-6, and tumour necrosis factor α (TNF-α) were higher in depressed patients than in healthy individuals (Alesci et al. 2005; Howren et al. 2009; Dowlati et al. 2010). Prospective studies have also shown that elevated levels of IL-6 in childhood significantly raise the risk of depression in adulthood (Khandaker et al. 2014). When inflammatory factors cross the BBB, the activities of 5-HT and DA neurons in the hypothalamus, hippocampus, and PFC are enhanced, thus increasing monoamine neurotransmitter reuptake in the CNS. Inflammatory factors also inhibit the utilization of tryptophan precursors and CRP associated with 5-HT synthesis, hence lowering neurotransmitter levels. Overexpression of inflammatory factors can also cause hyperactivation of the HPA and exacerbate depressive-like behaviour in rats (Koo et al. 2010). Furthermore, levels of CRP, IL-1β, TNF-α, and inflammatory molecules such as circulating leukocyte subsets might be used as predictors for the treatment of depression (Uher et al. 2014; Haapakoski et al. 2016).

## Iridoids in treatment of depression

**Figure 3.**
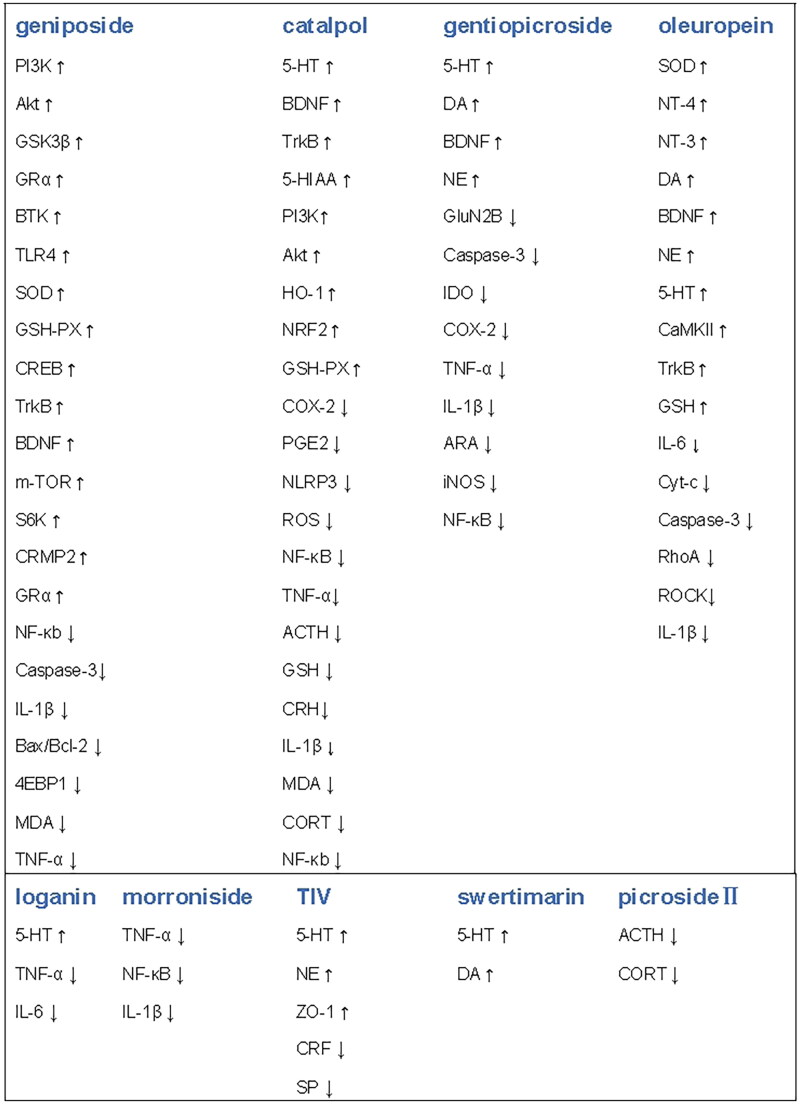
Major molecular target of iridoids and secoiridoids in prevention of depression. (↑)-denotes up-regulation, (↓)-denotes down-regulation.

The molecular targets and factors for the prevention of depression with iridoids and secoiridoids are shown in Figure 3.

### Geniposide

Recent studies have revealed that geniposide (GP) is one of the effective components in treating depression. Glucagon-like peptide-1 receptor (GLP-1R) activation triggers phosphorylation of PI3K, AKT, and glycogen synthase kinase 3 (GSK3), which improves mitochondrial function and reverses neuronal damage, apoptosis, and inflammation in the brain (Bassil et al. 2014). GP, a novel receptor agonist of GLP-1R, inhibits hippocampal neuronal apoptosis and reduces IL-1β and TNF-α levels in the repetitive restraint stress mice by activating GLP-1R/AKT to improve depression-like behaviour (Zhao et al. 2018). PI3K/Akt/GSK3β is a potential target and molecular mechanism for GP treatment of depression and activation of the PI3K/Akt signalling pathway can eliminate depressive-like behaviours mediated by repeated restraint stress in mice (Xian et al. 2019). The importance of PI3K/Akt/GSK3β has been demonstrated in animal experiments where administration of GP (30, 60, and 90 mg/kg, i.g.) to the chronic unpredicted mild stress (CUMS)-induced depression mice for 4 weeks significantly increased sucrose consumption in the sucrose preference test (SPT), the number of crossings in the OFT and shortened immobility time in the tail suspension test (TST) and the forced swimming test (FST). GP also alleviated hippocampal ceramide levels, attenuated hippocampal neuronal apoptosis, and enhanced the phosphorylated forms of PI3K, Akt, and GSK3β (Wang M et al. 2021). Overexpression of ceramide promotes neuronal apoptosis by inhibiting Akt/GSK3β signalling, and activation of PI3K/Akt signalling abrogates the CUMS-mediated depression-like behaviour in mice (Jazvinšćak Jembrek et al. 2015; Cai et al. 2020).

Another pivotal therapeutic target for antidepressant drug development is the mammalian target of rapamycin (m-TOR) pathway. In a rat model of the chronic mild stress (CMS) depression, the hippocampal m-TOR signalling pathway was significantly inhibited (Chen G et al. 2021). The expression of m-TOR, S6K, and collapsin response mediator protein 2 (CRMP2) in the PFC of Wistar rats increased following GP administration for 14 days. Bruton’s tyrosine kinase/Janus kinase/signal transducer and activator of transcription 1 (BTK/JAK2/STAT1) pathway exerts an antidepressant role in lipopolysaccharide (LPS)-depressed animals by modulating the polarization of microglia to the anti-inflammatory phenotype M2, and may be involved in the suppression of inflammatory response by GP (Zheng et al. 2021). The combination of GP and epimedoside B for adult male Institute of Cancer Research (ICR) mice was also associated with reduced levels of pro-inflammatory factors such as TNF-α and IL-1β. In addition, the NF-κB pathway is down-regulated (Zhang B et al. 2021). In the CUMS-induced model, upon administration of GP (10 or 40 mg/kg, i.g) in 7-week-old mice for 21 days, NF-κB level associated proteins were also down-regulated, superoxide dismutase (SOD) and glutathione peroxidase (GSH-PX) antioxidant activities were raised, and BTK, TLR4, and NF-κB associated proteins were down-regulated, whereas BDNF levels were up-regulated. BTK, TLR4, MyD88, and NF-κB protein levels were likewise decreased in corticosteroid-induced PC12 cells, according to *in vitro* investigations (Chen T et al. 2021). Normalization of BDNF expression in the hippocampus region by genipin, a product of GP hydrolysis by β-glucosidase, was achieved in a model of prenatal restraint stress mice by inhibiting DNA methyltransferase 1 (Ye et al. 2018). GP alleviates depression-like behaviour in the male Kunming (KM) mice model of streptozotocin (STZ)-induced diabetes mellitus combined with depression, which is similarly linked to increased BDNF expression in the brain (Wang J et al. 2016). In CUMS-induced rats, GP also reduced HPA axis hyperactivity and raised the number of glucocorticoid receptor α (GRα) immune cells in the paraventricular nucleus (Cai et al. 2015). GP therapy enhanced the activity of cAMP-response element binding protein (CREB) in the hippocampus tissue in a rat model of diabetes-related depression utilizing a high-fat diet supplemented with corticosterone (CORT), consistent with its pro-neurogenic effect (Sun B et al. 2021). In an *in vitro* model, GP improved neurite outgrowth in cultured Neuro2a cells inhibited by fluoxetine, and the combined treatment of the two raised the number of neurite cells by 3% compared with the fluoxetine group (Mkc et al. 2019). In another *in vitro* study, GP reversed C16 ceramide-induced apoptosis in primary hippocampal neurons from mice embryos and reduced the Bcl2-associated X/Bcl (Bax/Bcl-2) ratio and caspase-3 expression. In addition, PI3K, Akt, and GSK3β phosphorylation levels were increased (Wang M et al. 2021). In summary, GP appears to be very effective in improving depression.

### Catalpol

Roughly 32 iridoid compounds were identified from *Rehmannia glutinosa* roots, with catalpol being the most abundant, accounting for about 5.33% in undried and 0.61% in dried *Rehmannia glutinosa* roots (Liu et al. 2001). Catalpol has been reported to play a pivotal role in antidepressant. The PI3K/Akt/Nrf2/HO-1 pathway is possibly a potential biomarker and molecular target for catalpol antidepressants. Activation of the PI3K/Akt pathway can stimulate the activation of nuclear factor E2-related factor 2 (Nrf2)/heme oxygenase-1 (HO-1) pathway, which binds antioxidant response elements and thus protects the body from oxidative stress (Yuan et al. 2020). In the CUMS-induced depressed Sprague Dawley (SD) rat model, the expressions of PI3K, Akt, Nrf2, HO-1, TrkB, and BDNF in the hippocampus were decreased. The abnormality of the above indexes was observably reversed 5 weeks following catalpol therapy (10 mg/kg, i.g.), and the effect of catalpol and fluoxetine was equivalent (Wang J et al. 2021). Catalpol alleviates depression-like behaviour in the STZ-induced hyperglycaemia animal model, which is similarly associated with the PI3K/Akt/Nrf2/HO-1 pathway. The anomalies in TST, FST, and OFT were significantly reversed after administering catalpol (5, 10, or 20 mg/kg) orally for 21 days. In the hippocampus and PFC, catalpol also restored abnormal PI3K and AKT, as well as abnormal levels of the Nrf2 protein, HO-1, and antioxidants such as SOD, GSH-PX, reduced glutathione (GSH), and malondialdehyde (MDA) (Wu et al. 2021).

Cyclooxygenase-2 (COX-2) is another crucial biomarker in the treatment of depression and COX-2 inhibitors are protective against depression (Mueller 2010). Once cells are stimulated by inflammation, the expression level of COX-2 can be increased to 10–80 times of the normal level, resulting in the increase of PEG2, PGI2, and PGE1 in the inflammatory site, triggering an inflammatory response and tissue damage (Zhou et al. 2009). In the CUMS depression model of male SD rats, catalpol treatment (5, 10, or 20 mg/kg, i.g.) elevated BDNF activity and TrkB expression, lowering COX-2 and its downstream product PGE2 and normalizing the HPA axis by lowering serum CORT levels (Wang et al. 2015). Catalpol also inhibited HPA axis hyperactivity in a model of CORT-induced depression (Song et al. 2021). Recovery of HPA axis abnormalities in adult male KM mice after catalpol administration and significant reduction in serum CORT, ACTH, and CRH levels and NF-κB phosphorylation levels in hippocampus and frontal cortex, and upregulation of Nrf2 expression levels. Activation of NF-κB, a key regulator of gene transcription of inflammatory cytokines, upregulates the expression of inflammatory factors such as TNF-α and IL-1β, and plays a critical role in the pathogenesis of depression (Camargo et al. 2020; Huang X et al. 2020).

The NLRP3 inflammatory complex is a molecular mechanism that converts psychological stress stimuli into an inflammatory response, and its activation may trigger the pathogenesis of depression (Zhang Y et al. 2015). In CUMS-mediated male C57BL/6 depressive mice, catalpol (20 mg/kg) inhibited NLRP3 inflammatory activation, pro-inflammatory cytokines, and reactive oxygen species (ROS) (Wang Y et al. 2021; Wang YL et al. 2021). Catalpol (5, 10, or 20 mg/kg, i.g.) administration significantly counteracted reserpine-induced rectal temperature drop, dyskinesia, and ptosis, as well as increasing 5-HT and its metabolite 5-hydroxyindoleacetic acid (5-HIAA) levels in the brains of mice, but had no effect on NE or DA levels. The antidepressant effect of catalpol was possibly mediated by the central 5-HIAA system, rather than the NE or DA systems (Wang J et al. 2014). By enhancing neuronal differentiation and survival of mature neurons, as well as promoting exercise-mediated hippocampus neurogenesis, catalpol improved sadness, anxiety, and cognitive capacity of 8 weeks adult C57BL/6N male mice in post-traumatic stress disorder (Sun L et al. 2021). In summary, catalpol exerts a multifaceted antidepressant effect by increasing brain 5-HT levels, lowering oxidative stress, and restoring hyperfunction of the HPA axis. However, no clinical studies have currently been conducted to determine if catalpol is effective against depression in humans.

### Loganin

Loganin is an iridoid glycoside that has antidepressant properties and is found in *Cornus officinalis* (Loganaceae). Loganin has been proved to reach the brain through the BBB (Li X et al. 2008). Microglia is potentially a therapeutic key of loganin for depression (Kalkman and Feuerbach 2016; Zhang L et al. 2018). In patients with depression, peripheral inflammation drives microglia into a pro-inflammatory phenotype and causes microglia M1 polarization, resulting in more severe neuroinflammation. Hence, inhibition of microglia-induced neuroinflammation improves depression-like behaviour (Chan et al. 2019; Deng et al. 2020). By using cultured BV-2 microglia and an LPS-induced inflammation model, loganin was discovered to lessen inflammation by suppressing M1 polarization in microglia. According to network pharmacology, catenin Beta 1 (CTNNB1) is predicted to be a major node in the loganin-information-depression crossover network, with Th1 and Th2 cell differentiation and the IL-17 signalling pathway linked to depression (Xia et al. 2021). In addition, loganin administration (12.5, 50 mg/kg) for 3 days significantly reduced resting time in the TST of adult male C57BL/6 mice and improved reserpine-induced hypothermia and ptosis. The *in vivo* experiments showed that increased 5-HTP levels (a precursor of 5-HT) induced head twitch responses, as well as significantly increased 5-HT levels in the PFC, hippocampus, and striatum (Pan et al. 2021). Treatment with loganin (40 mg/kg) for 10 days in the adult male Wistar rat model of depression and diabetes significantly lowered serum concentrations of IL-6 and TNF-α, shortened immobility in the FST, and restored weight growth and blood glucose alterations (Rajabi et al. 2018). Currently, research on the treatment of depression with loganin is scarce and still in its initial stages, so there is plenty of room for development.

### Morroniside

Morroniside is also a naturally occurring iridoid glycoside isolated from the traditional medicinal plant *Cornus officinalis* Siebold & Zucc. (Cornaceae) (Lei et al. 2018). Morroniside may be useful in treating depression by inhibiting the NF-κB signalling pathway and reducing the expression of inflammatory proteins, hence lowering the neuroinflammatory response. A recent study showed that CUMS-induced depressive symptoms were significantly improved in adult SD rats treated with morroniside (200 mg/kg, i.g.) for 10 days. IL-1β, TNF-α, and NF-κB levels were reduced and pathological damage was reversed (Wei 2020).

### TIV

The roots and rhizomes of *Valeriana jatamansi* Jones (VJJ) (Valerianaceae) have been used in the treatment of nervous system diseases in traditional Chinese medicine for thousands of years (Tang et al. 2002). VJJ can considerably enhance the mental condition of people and contains a variety of chemical components such as iridoids, alkaloids, and volatile oil. Among them, total iridoids of VJJ (TIV) are the main sedative component of VJJ (Lin et al. 2009; Sah et al. 2011). TIV has been demonstrated to be effective in treating depression, and multiple studies have revealed that valepotriate is the major antidepressant component of TIV (Müller et al. 2015; Jugran et al. 2019). After 2 weeks of treatment with TIV (5.7, 11.4, or 22.9 mg/kg) in CUMS-induced depressed adult KM male rats, the body weight and sucrose consumption in SPT were increased, the expression levels of 5-HT and NE in the hippocampus and colon were elevated, and the expression levels of SP and corticotropin releasing factor (CRF) were decreased (Wang L et al. 2020). In addition, administration of TIV for 2 weeks in CUMS adult male KM mice could also elevate the expression of zonula occludens-1 (ZO-1) and occludin to reduce the BBB permeability and balance the relative abundance of firmicutes and bacteroidetes (Zhang L et al. 2021). According to a comprehensive serum metabolomics study using NMR, TIV may exert antidepressant effects through various metabolic pathways, including neurotransmitter synthesis, tricarboxylic acid cycle regulation, and amino acid metabolism (Li, Wu, et al. 2020). The utilization of metabolomic approaches to screen and identify depression-related biomarkers may contribute to a better understanding and treatment of depression.

### Oleuropein

Oleuropein (OE), a secoiridoid glycoside broad existing in Oleaceae plants, has a wide range of pharmacological effects, including anti-inflammatory, antiatherosclerosis, anticancer, and antibacterial properties. The absorption rate of OE in humans is about 50–60% and the active metabolites of OE can cross the BBB and act on the brain (Omar 2010; Carito et al. 2014). BDNF/TrkB might be a critical target for OE treatment of depression. By establishing a SH-SY5Y cell model, the expression of BDNF and TrkB increased after OE treatment combined with BDNF overexpression and expression levels were further raised after BDNF overexpression. BDNF/TrkB is speculated to cause an increase in serum calcium ion concentration in depressed C57/BL mice through activation of CaMKII signalling to alleviate depressive symptoms. After treatment, expression of the Ras homolog gene family member A/Rho-associated protein kinase (RhoA/ROCK) was down-regulated, NT-3 and NT-4 was enhanced, and the inflammatory response to LPS stimulation was reduced. Furthermore, the immobility time in TST, OFT, and FST was increased *in vivo*, and IL-1β, IL-6 and other inflammatory factors were reduced (Nie and Xu 2021). The antioxidant capacity and citrate synthase activity of high-fat diet (HFD) induced C57BL/6J mice with depression were increased after administration of OE (100 mg/kg, i.g.) for 10 weeks, but hippocampal BDNF mRNA did not also increase (Mikami et al. 2021). These results are inconsistent with the study by Nie and Xu (2021). An *in vivo* study revealed that NE, 5-HT, DA, and GSH levels in the brain of depressed rats were upregulated by OE and LPO was inhibited by OE. By comparing the antidepressant effects at different doses (5, 10, and 20 mg/kg), it was found that OE prevented depression at all of the administered dose levels, but the best effect was seen at 20 mg/kg (Badr et al. 2020). OE also reduced cell oxidative stress damage in the hydrogen peroxide (H_2_O_2_)-induced PC12 cell model by suppressing cell viability loss, reducing apoptosis rate, inhibiting the decrease of intracellular SOD, and up-regulating the expression of apoptotic proteins Cyt-c and caspase-3 (Li and Li 2019).

### Gentiopicroside

Gentiopicroside (Gent) is a secoiridoid glycoside identified from *Gentiana manshurica* Kitagawa, *Gentiana scabra* Bunge, or *Gentiana triflora* Pall (Gentianaceae). Recently, Gent has been reported as an antidepressant for reducing inflammation and pain (Wu S et al. 2017). GluN2B is an essential target for Gent in the treatment of depression. NMDA (GluN2B-NMDA) receptors containing the GluN2B subunit and the NMDA receptor antagonist, ketamine, have been shown to rapidly improve symptoms of depression (Debacker et al. 2015; Zhang W et al. 2018). A study demonstrated that Gent may inhibit reserpine-induced pain and depression by downregulating GluN2B-containing NMDA receptors in the amygdala, and administration of Gent (50 mg/kg, i.p.) for three days significantly increased the levels of NE, DA, and 5-HT in the amygdala and down-regulated caspase-3 (Liu et al. 2014). In LPS-induced male BALB/C mice, Gent also down-regulated the activation of tryptophan metabolic pathways in mouse brain tissue and the expression of GluN2B in the PFC. Gent additionally prevented the over-activation of indoleamine 2,3-dioxygenase (IDO) and reduce TNF-α and IL-1β levels in the BLA and PFC. Hence, Gent is possibly a multi-targeted antidepressant that operates by inhibiting different phases of the pro-inflammatory cytokine-induced tryptophan degradation pathway (Deng et al. 2018). Gent attenuated elevated levels of arachidonic acid and slowed hippocampus apoptosis in a CORT-induced 5-week SD depression model. By inhibiting arachidonic acid (ARA) levels, Gent may protect cells from apoptosis and oxidative damage (Yao et al. 2019). *In vitro*, Gent down-regulated the expression of iNOS and COX-2, alleviated the damage of activated astrocytes to neurons (Deng 2015) and increased the proliferation of nerve cells and the level of BDNF (Yao et al. 2019). Therefore, Gent can also be utilized as an antidepressant.

### Swertimarin

Swertimarin, one of the main components in *Enicostemma littorale* Blume (Gentianaceae), has the characteristics of being analgesic, neuroprotective, anti-inflammatory, antiarthritic, hepatoprotective, antioxidative, and antibacterial (Fadzil et al. 2021). Swertiamarin was utilized as an antidepressant in mice and rats in previous investigations, but the exact mechanism has yet to be reported (Bhattacharya et al. 1976). 5-HT2 antagonists have been reported to be antidepressants with effects on both 5-HT and DA levels (Moreau et al. 1996). A recent study has shown that swertimarin has similar pharmacological effects to 5-HT2 antagonists (Sonawane et al. 2015). Thereby, swertiamarin possibly exerts antidepressant effects by modulating 5-HT and DA levels.

### PicrosideII

Picroside II is the key active ingredient of *Picrorhiza scrophulariiflora* Pennell (Scrophulariaceae) with a catechol-based structure (Li Q et al. 2010). The mechanism of picroside II in the treatment of depression is speculated to be associated with the antagonism of HPA axis hyperfunction and the regulation of ACTH and CORT levels in chronic stress rats. A recent study has shown that picroside II alleviates the depressive behaviour damage of CMS rats. The immobility duration of FST was reduced after 14 days of treatment with picroside II (1, 3, 10 mg/kg), the crossing and rearing scores of OFT were improved, along with reduced concentrations of ACTH and CORT (Zhou and Xiong 2011).

## Bioavailability of iridoids and glycosides in the brain

Neuro-related diseases, such as depression, are related to brain neurons. To treat encephalopathy, drugs must travel through the brain to play their therapeutic role. Therefore, the bioavailability of neuroprotective compounds in the brain such as iridoids and secoiridoids and their glycosides should be measured in detail to improve drug efficacy. Due to the BBB, the drug concentration in brain tissue after traditional administration is usually quite low, which seriously weakens the therapeutic effect of medications. A Study found that the selective permeability of iridoid glycosides and secoiridoid glycosides through BBB depends on their lipophilicity (Stefani and Rigacci 2014). Generally, compared with their carbohydrates, aglycones and acylated iridoids are more likely to passively diffuse through BBB. Numerous studies revealed that geniposide, oleuropein, catalpol, loganin, and picrosideII can cross the BBB and enter the CNS to exert a neuroprotective role (Wang F et al. 2014; Xue et al. 2015; Dinda et al. 2016). However, swertiamarin has difficulty crossing the BBB (Xu et al. 2013). Pentaacetyl geniposide ((Ac) 5GP), an acetylated derivative of geniposide, was found to have better lipophilicity and antidepressant effect than its parent geniposide (Peng et al. 2006; Cai et al. 2020). A recent study found that nasal administration can deliver exogenous substances directly from the nasal cavity to the brain, typically named a nasal-to-brain drug delivery (NBDD) (Miyake and Bleier 2015). Compared with intraperitoneal injection, nasal administration significantly increased geniposide concentration in the mice’s brain, and geniposide coupled with natural borneol showed faster absorption and slower elimination rate (Lu et al. 2012). NBDD of catalpol, showed strong brain targeting as the brain targeting index was greater than 1. It also increased SOD activity and decreased MDA activity by up-regulating the expression of Nrf2 and HO-1, minimizing oxidative stress injury (Wang J et al. 2022). NBDD might be a potential and promising route of administration for the treatment of brain-related diseases in the future.

In addition, geniposide-solid lipid nanoparticles (SLNs) are a promising delivery system, with a study showing that SLNs production can assist improve bioavailability (Wang F et al. 2014). González et al. (2020) developed a strategy for embedding iridoid compounds by spray drying olive leaf extract with maltodextrin or inulin to improve the bioavailability of OE. Inhibiting enzymatic hydrolysis *in vivo* also enhanced bioavailability, according to a study by Dai et al. (2016). The relative oral bioavailability of d-cellobiose was improved by 72.13% (1:5) and 106.3% (1:10) when compared to the gentiopicroside group. To overcome the problem that exogenous BDNF does not directly cross the BBB, the researcher has developed a liposome that can carry exogenous BDNF through the BBB. In addition, phospholipid complex, microemulsion, or gel polymer micelles could be used to improve the pharmacokinetic characteristics of drugs and improve their oral bioavailability.

## Plant sources of the antidepressant iridoids and secoiridoids

The important plant sources and related references of iridoids and secoiridoids are listed in Table 2. This comprehensive summary will contribute to identifying these plant metabolites in large quantities for future study in this field.

**Table 2.** List of major plant sources and isolation references of iridoids against depression.

Compound	Plant sources	Reference(s)
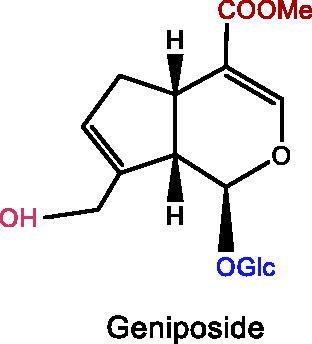	*Gardenia jasminoides Rehmannia glutinosa Eucommia ulmoides Cistanche deserticola Achyranthes bidentata Genipa americana Genipap fruit Randia spinosa*	(Hamerski et al. 2003; Ono et al. 2005; Nathia-Neves et al. 2017; Shan et al. 2017)
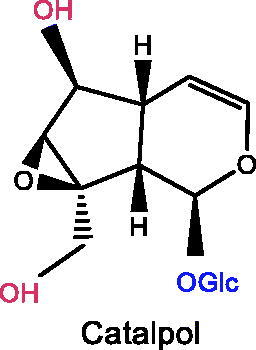	*Rehmannia glutinosa Centranthera grandiflora Buddleia globosa Plantago argentea Centranthera grandiflora Lagotidis* Herb*Veronica longifolia Radix Scrophulariae Lanceatibetica*	(Duff et al. 1965; Suomi et al. 2000; Sertic et al. 2015; Wang et al. 2016; Bai et al. 2019; Wan et al. 2019; Zhang et al. 2020; He et al. 2021)
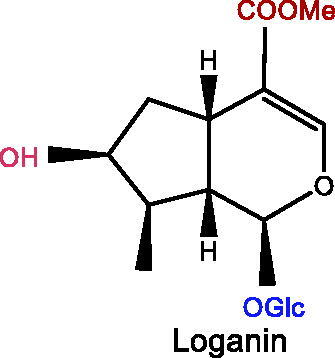	*Cornus officinalis Palicourea rigida Parentucellia latifolia Lonicera japonica Haskap* berry*Kirengeshoma palmata Scabiosa variifolia Strynos nux-vomica Cornus mas*	(Yamamoto et al. 1999; Papalexandrou et al. 2003; Khan et al. 2012; da Silva et al. 2013; Dinda et al. 2016; Gousiadou et al. 2016; Lei et al. 2018; Llorent-Martínez et al. 2019; Martinez et al. 2021)
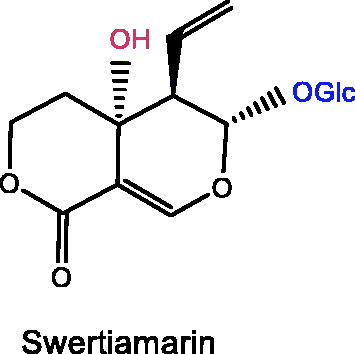	*Gentiana manshurica Gentiana macrophylla Gentiana scabra Gentiana triflora Swertia tetraptera Gentiana lutea*	(Jiang et al. 2003; Öztürk et al. 2006; Jiang et al. 2012; Kuang et al. 2016; Choi et al. 2019; Huang et al. 2020)
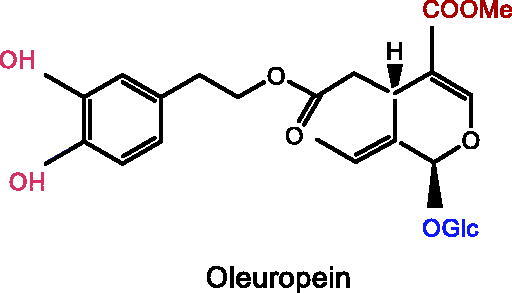	*Olea europaea Syringa pubescens Syringa microphylla Ligustrum lucidum Jasminum officinale*	(Papalexandrou et al. 2003; Zhao et al. 2009; Wang et al. 2020; Liu et al. 2021; Yang et al. 2021)
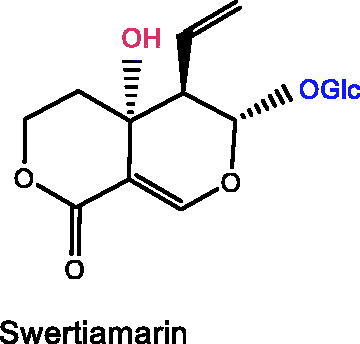	*Enicostemma littorale Scabiosa variifolia Centaurium erythraea Eustoma grandiflorum Fragrea fragrans Gentiana algida Swertia angustifolia Swertia tetraptera*	(Anwar et al. 1996; Papalexandrou et al. 2003; Jiang et al. 2012; Fadzil et al. 2021)
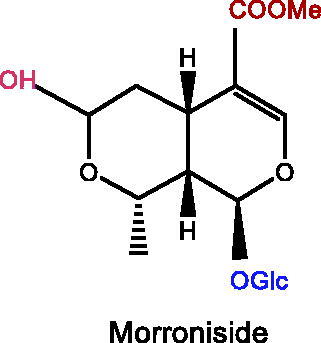	*Cornus officinalis Gentiana straminea*	(Lei et al. 2018; Zhou et al. 2021)
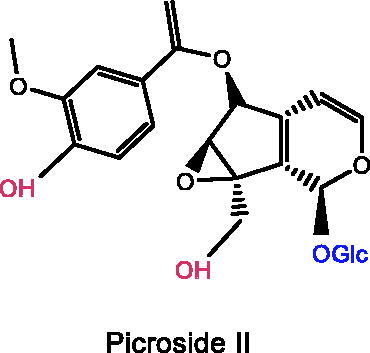	*Picrorhiza scrophulariiflora Picrorhiza kurrooa*	(Li et al. 1998)
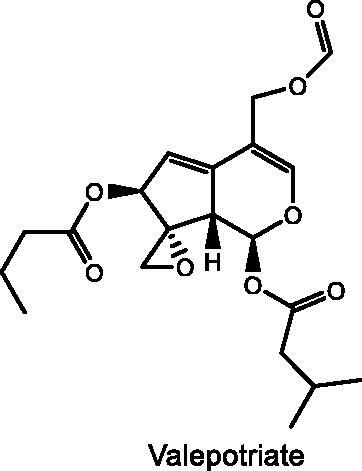	*Valeriana jatamansi Valeriana glechomifolia Valeriana laurifolia Valeriana officinalis Valeriana amurensis Valeriana polystachya*	(Giraldo et al. 2013; Müller et al. 2015; Wu et al. 2017)

## Conclusions and future perspectives

The treatment of mental disorders such as depression, which affects hundreds of millions of people around the world, is a difficult issue for both basic science and clinical medicine. The drugs currently in clinical use still do not cure these disorders and have a relapse rate of up to 80%. Disturbances in the neuroendocrine system, immune system, and metabolic levels caused by stress may underlie the pathology of depression. Chronic stress raises the activity of the HPA axis and GC secretion, which influences the release of pro-inflammatory cytokines including IL-1β and TNF-α. The active ingredients of the classical Chinese antidepressant formulae, iridoids, improve depression with multi-target therapy. Iridoids compounds including GP, catalpol, Gent, OE, loganin, swertimarin, and TIV all alleviate depression by raising levels of monoamine neurotransmitters such as 5-HT, DA, and NE. GP, catalpol, Gent, and OE reduce depression by elevating the expression of BDNF and its receptor TrkB. Catalpol, Gent, and OE inhibit the overexpression of the HPA axis. Furthermore, GP, catalpol, and picroside II reduce depressive symptoms by suppressing the release of hormones such GRα, ACTH, and CRH. Hence, it is necessary to create more effective multi-targeted therapeutics involved in the subsequent diagnosis and treatment of depression, including targeting the serotonergic system, NE system, neuroimmune system, neurotrophic factors, and neuroendocrine system.

With the advent of molecular biology, several clinical biomarkers and molecular targets of depression have been identified. However, due to the complexity and heterogeneity of depression and its correlation with other co-morbid psychiatric disorders, we are still in the dark for identifying specific biomarkers and molecular targets to stop or mitigate the progression of depression at different stages. Metabolomics is a research method used to comprehensively examine the biochemical changes occurring in the body, to explore their underlying pathophysiological mechanisms, and to assess the efficacy of drugs (Dai et al. 2010). Metabolic disorders are associated with depression, and a total of 18 different metabolites have been identified in normal rats, depressed rats, and gentiopicroside-treated depression-like rats. Among them, ARA, oxoadipic acid, l-phenylalanine, and thiamine were expressed at high levels in depressed rats, while sphingosine, stearoylethanolamide, guanosine, and acetic acid, were present at low levels in depressed rats and their expression was significantly restored after gentiopicroside administration (Yao et al. 2019). At present, there are few relevant studies on the metabolomic of iridoids for the treatment of depression. Thus, metabolomic studies to identify metabolites differentially expressed in different treatment groups in subsequent studies may be a critical direction.

Our findings from reported data demonstrate that a variety of iridoids are the active ingredients of several antidepressant formulae and have strong intracerebral targeting properties and therefore have great potential to be developed as antidepressants. However, there are still some issues to be resolved. The specific mechanism of action targets of many iridoids is not known, and further studies are needed to elucidate the detailed mechanisms. Although several iridoids have shown significant efficacy in stressed animal models as well as exogenous drug-induced models, they have not been tested in genetic animal models and transgenic animals, which is a major research limitation. In subsequent studies, experiments should be conducted by breeding experimental animals for depression preferences through innate genetic specificity or knocking out specific genes. Examples to obtain more insightful results include the Flinders sensitive line rat model, the Holtzman Albino strain rat model, and the Tryon Maze Dull rat model. Above all, no human clinical trials have been conducted. Another issue to resolve is that iridoid compounds are unstable and degraded under physical and chemical conditions, which hamper the study of their activity and function, and monomer research is relatively limited. Iridoids also have a low cerebral distribution, rapid absorption and elimination *in vivo*, and low bioavailability for oral administration. Hence, future research should focus on improving iridoids’ extraction and purification methods, researching pharmacokinetics in brain tissues to enhance bioavailability, conducting metabolomics studies to explore potential physiological mechanisms, determining effective doses, structural modifying toxic compounds or developing new dosage forms, and furthering the study of key molecular targets. Such research must be accomplished before iridoids can be subjected to large-scale clinical trials in humans and used as prospective or lead drugs in the development of more effective therapeutics. In addition to iridoids, more potential compounds in natural products should be further studied for treating depression.

## Authors’ contributions

Yaoyao Kou designed the structure and edited the manuscript. Zhihao Li is responsible for the figures. Tong Yang, Xue Shen, and Xin Wang proofread the manuscript. Kun Zhou, Luyao Li, and Zhaodi Xia systemically revised the manuscript for critical content. Ye Zhao and Xiaohui Zheng proposed the concept and designed the structure of the manuscript.
